# Supplementary material for: The Effects of Fruit-Derived Polyphenols on Cognition and Lung Function in Healthy Adults: A Systematic Review and Meta-Analysis
Source: Nutrients. 2021 Nov 27;13(12):4273. doi: 10.3390/nu13124273 (PMC8708719; doi:10.3390/nu13124273)
Supplement: Supplementary file 1 [file nutrients-13-04273-s001.zip › Cognition- Summary NIH Quality Assessment Tool for Controlled trials.pdf]

Table S1: Cognition - NIH Quality Assessment Tool for Controlled Trials

Criteria	1*	2	3	4	5	6	7	8	9	10	11	12	13	14	15
1. Was the study described a randomised trial, a randomised clinical trial, or an RCT?	N	Y	Y	Y	Y	Y	Y	Y	Y	Y	Y	Y	Y	Y	Y
2. Was the method of randomization adequate (i.e., use of randomly generated assignment)?	N	NR	Y	Y	NS	NS	NS	Y	N	Y	NS	NS	NS	Y	Y
3. Was the treatment allocation concealed (so that assignments could not be predicted)?	N	Y	Y	Y	Y	NR	Y	NS	Y	Y	N	Y	Y	Y	NR
4. Were study participants and providers blinded to treatment group assignment?	N	Y	Y	Y	Y	Y	Y	N	Y	Y	N	Y	Y	Y	NR
5. Were the people assessing the outcomes blinded to the participants; group assignments?	N	Y	Y	Y	Y	N	Y	N	Y	N	N	Y	N	N	N
6. Were the groups similar at baseline on important characteristics that could affect outcomes?	Y	Y	Y	NR		NR	Y	Y	NS	Y	NS	NS	Y	N	Y
7. Was the overall drop-out rate from the study at endpoint 20% or lower of the number allocated to treatment?	Y	Y	Y	N	Y	Y	Y	Y	Y	Y	Y	Y	Y	Y	Y
8. Was the differential drop-out rate (between treatment groups) at endpoint 15 percentage points or lower?	Y	Y	Y	NS	Y	Y	Y	Y	Y	Y	Y	Y	Y	Y	Y
9. Was there high adherence to the intervention protocols for each treatment group?	NS	NS	Y	NS	Y	Y	NS	NS	Y	NS	Y	Y	Y	Y	NS
10. Were other interventions avoided or similar in the groups (e.g. similar background treatments)?	NS	NS	Y	N	Y	Y	Y	Y	NS	Y	Y	Y	Y	Y	NS
11. Were outcomes assessed using the most valid and reliable measures, implemented consistently across all study participants?	Y	Y	Y	NR	Y	Y	Y	Y	Y	Y	Y	Y	Y	Y	Y
12. Did the authors report that the sample size was sufficiently large to be able to detect a difference in the main outcome between groups with at least 80% power?	N	Y	Y	Y	Y	NS	Y	N	N	N	N	Y	N	N	N
13. Were outcomes reported or subgroups analysed pre-specified (i.e. identified before analyses were conducted)?	Y	NS	NR	NS	Y	NS	NS	Y	Y	Y	Y	Y	Y	Y	NS
14. Were all randomised participants analysed in the group to which they were originally assigned?	Y	Y	Y	Y	Y	Y	Y	Y	Y	Y	Y	Y	Y	Y	Y

* denotes lung study, Y = Yes, N = No, NR = Not Really, NS = Not Stated.

1. Vergara D, Avila D, Escobar E, Carrasco-Pozo C, Sanchez A, Gotteland M. The intake of maqui (aristotelia chilensis) berry extract normalizes H2O2 and IL-6 concentrations in exhaled breath condensate from healthy smokers - an explorative study. *Nutrition Journal*. **2015**;14:27.

2. Bowtell JL, Aboo-Bakkar Z, Conway ME, Adlam AR, Fulford J. Enhanced task-related brain activation and resting perfusion in healthy older adults after chronic blueberry supplementation. *Appl Physiol Nutr Metab dnlm*. **2017**;42(7):773-779. doi: 10.1139/apnm-2016-0550.

3. Bell L, Whyte AR, Lamport DJ, Spencer JP, Butler LT, Williams CM. Grape seed polyphenol extract and cognitive function in healthy young adults: A randomised, placebo-controlled, parallel-groups acute-on-chronic trial. *Nutr Neurosci*. **2020**:1-10

4. Gibson N, Baker D, Sharples A, Braakhuis A. Improving mental performance in an athletic population with the use of ārepa ® , a blackcurrant based nootropic drink: A randomized control trial. *Antioxidants*. **2020**;9(4):316. doi: 10.3390/antiox9040316.

5. Keane KM, Haskell-Ramsay CF, Veasey RC, Howatson G. Montmorency tart cherries (prunus cerasus L.) modulate vascular function acutely, in the absence of improvement in cognitive performance. *Br J Nutr*. 2016;116(11):1935-1944.

6. Whyte AR, Cheng N, Butler LT, Lamport DJ, Williams CM. Flavonoid-rich mixed berries maintain and improve cognitive function over a 6 h period in young healthy adults. *Nutrients*. **2019**;11(11):2685.

7. Alharbi M, Lamport D, et al. Flavonoid-rich orange juice is associated with acute improvements in cognitive function in healthy middle-aged males. *Eur J Nutr*. **2016**;55(6). doi: 10.1007/s00394-015-1016-9.

8. Nilsson A, Salo I, Plaza M, Björck I. Effects of a mixed berry beverage on cognitive functions and cardiometabolic risk markers; A randomized cross-over study in healthy older adults. *PloS one*. **2017**;12(11)

9. Watson AW, Okello EJ, Brooker HJ, Lester S, McDougall GJ, Wesnes KA. The impact of blackcurrant juice on attention, mood and brain wave spectral activity in young healthy volunteers. *Nutr Neurosci*. **2018**;22(8):596-606. doi: 10.1080/1028415x.2017.1420539.

10. Philip P. Acute intake of a grape and blueberry polyphenol-rich extract ameliorates cognitive performance in healthy young adults during a sustained cognitive effort. *Antioxidants.* **2019**;8(12):650.

11. Cook MD, Sandu B, Amber Kaur, Joyce P, Jennifer Patricia. Effect of New Zealand blackcurrant on blood pressure, cognitive function and functional performance in older adults. *Journal of Nutrition in Gerontology and Geriatrics*. **2020**;39(2):99-113.

12. Jackson PA, Wightman EL, Veasey R, et al. A randomized, crossover study of the acute cognitive and cerebral blood flow effects of phenolic, nitrate and botanical beverages in young, healthy humans. *Nutrients*. **2020**;12(8):2254.

13. Khalid S, Barfoot KL, May G, Lamport DJ, Reynolds SA, Williams CM. Effects of acute blueberry flavonoids on mood in children and young adults. *Nutrients*. **2017**;9(2):158.

14. Miller MG, Hamilton DA, Joseph JA, Shukitt-Hale B. Dietary blueberry improves cognition among older adults in a randomized, double-blind, placebo-controlled trial. *Eur J Nutr*. **2018**;57(3):1169-1180.

15. Igwe EO, Roodenrys S, Probst YC, et al. Low anthocyanin plum nectar does not impact cognition, blood pressure and gut microbiota in healthy older adults: A randomized crossover trial. *Nutrition Research*. **2020**;82:74-87. doi: 10.1016/j.nutres.2020.08.003.
