# Supplementary material for: The Effects of Fruit-Derived Polyphenols on Cognition and Lung Function in Healthy Adults: A Systematic Review and Meta-Analysis
Source: Nutrients. 2021 Nov 27;13(12):4273. doi: 10.3390/nu13124273 (PMC8708719; doi:10.3390/nu13124273)
Supplement: Supplementary file 1 [file nutrients-13-04273-s001.zip › Lung - Summary NIH Quality Assessment Tool for Observational Cohort and Cross.pdf]

Table S2: NIH Quality Assessment Tool for Observational Cohort and Cross-Sectional Studies

Lung

Criteria	1	2	3	4	5	6
1. Was the research question or objective in this paper clearly stated?	Y	?	NR	Y	Y	Y
2. Was the study population clearly specified and defined?	Y	Y	Y	Y	Y	Y
3. Was the participation rate of eligible persons at least 50%?	Y	Y	Y	Y	Y	Y
4. Were all the subjects selected or recruited from the same or similar populations (including the same time period)? Were inclusion and exclusion criteria for being in the study prespecified and applied uniformly to all participants?	Y	?	Y	Y	Y	Y
5. Was a sample size justification, power description, or variance and effect estimates provided?	N	Y	N	N	N	N
6. For the analyses in this paper, were the exposure(s) of interest measured prior to the outcome(s) being measured?	Y	N	N	Y	Y	Y
7. Was the timeframe sufficient so that one could reasonably expect to see an association between exposure and outcome if it existed?	Y	N	NA	NA	NA	Y
8. For exposures that can vary in amount or level, did the study examine different levels of the exposure as related to the outcome (e.g., categories of exposure, or exposure measured as continuous variable)?	Y	Y	Y	Y	N	N
9. Were the exposure measures (independent variables) clearly defined, valid, reliable, and implemented consistently across all study participants?	Y	Y	Y	Y	Y	Y
10. Was the exposure(s) assessed more than once over time?	N	N	Y	N	N	N
11. Were the outcome measures (dependent variables) clearly defined, valid, reliable, and implemented consistently across all study participants?	Y	Y	Y	Y	Y	Y
12. Were the outcome assessors blinded to the exposure status of participants?	Y	Y	Y	Y	Y	N
13. Was loss to follow-up after baseline 20% or less?	?	NA	Y	NA	NA	Y

14. Were key potential confounding variables measured and adjusted statistically for their impact on the relationship between exposure(s) and outcome(s)?	Y	y	Y	Y	Y	N

1. Mehta AJ, Cassidy A, Litonjua AA, Sparrow D, Vokonas P, Schwartz J. Dietary anthocyanin intake and age-related decline in lung function: Longitudinal findings from the VA normative aging study. *Am J Clin Nutr*. **2016**;103(2):542-550.
2. Garcia-Larsen V, Thawer N, Charles D, et al. Dietary intake of flavonoids and ventilatory function in European adults: A GA2LEN study. *Nutrients*. **2018**;10(1).
3. Butland BK, Fehily AM, Elwood PC. Diet, lung function, and lung function decline in a cohort of 2512 middle aged men. *Thorax*. **2000**;55(2):102-108.
4. Pounis G, Arcari A, Costanzo S, et al. Favorable association of polyphenol-rich diets with lung function: Cross-sectional findings from the Moli-Sani study. *Respir Med*. **2018**;136:48-57.
5. Tabak C, Arts IC, Smit HA, Heederik D, Kromhout D. Chronic obstructive pulmonary disease and intake of catechins, flavonols, and flavones: The MORGEN study. *American Journal of Respiratory and Critical Care Medicine*. **2001**;164(1):61-64.
6. Vergara D, Avila D, Escobar E, Carrasco-Pozo C, Sanchez A, Gotteland M. The intake of maqui (aristotelia chilensis) berry extract normalizes H2O2 and IL-6 concentrations in exhaled breath condensate from healthy smokers - an explorative study. *Nutrition Journal*. **2015**;14:27.
